# Effects of polyploidy and reproductive mode on life history trait expression

**DOI:** 10.1002/ece3.1934

**Published:** 2016-01-11

**Authors:** Katelyn Larkin, Claire Tucci, Maurine Neiman

**Affiliations:** ^1^ Department of BiologyUniversity of IowaIowa CityIowa52242

**Keywords:** Asexuality, genomic variation, mating system, organismal ecology, phenotypic variation, ploidy level

## Abstract

Ploidy elevation is increasingly recognized as a common and important source of genomic variation. Even so, the consequences and biological significance of polyploidy remain unclear, especially in animals. Here, our goal was to identify potential life history costs and benefits of polyploidy by conducting a large multiyear common garden experiment in *Potamopyrgus antipodarum*, a New Zealand freshwater snail that is a model system for the study of ploidy variation, sexual reproduction, host–parasite coevolution, and invasion ecology. Sexual diploid and asexual triploid and tetraploid *P. antipodarum* frequently coexist, allowing for powerful direct comparisons across ploidy levels and reproductive modes. Asexual reproduction and polyploidy are very often associated in animals, allowing us to also use these comparisons to address the maintenance of sex, itself one of the most important unresolved questions in evolutionary biology. Our study revealed that sexual diploid *P. antipodarum* grow and mature substantially more slowly than their asexual polyploid counterparts. We detected a strong negative correlation between the rate of growth and age at reproductive maturity, suggesting that the relatively early maturation of asexual polyploid *P. antipodarum* is driven by relatively rapid growth. The absence of evidence for life history differences between triploid and tetraploid asexuals indicates that ploidy elevation is unlikely to underlie the differences in trait values that we detected between sexual and asexual snails. Finally, we found that sexual *P. antipodarum* did not experience discernable phenotypic variance‐related benefits of sex and were more likely to die before achieving reproductive maturity than the asexuals. Taken together, these results suggest that under benign conditions, polyploidy does not impose obvious life history costs in *P. antipodarum* and that sexual *P. antipodarum* persist despite substantial life history disadvantages relative to their asexual counterparts.

## Introduction

There is a growing body of evidence that polyploidy has played a key role in the diversification of angiosperms (*e.g*., *Amborella* Genome Project 2013; Vanneste et al. 2014) and other eukaryotic taxa (Van de Peer et al. 2009). Increased recognition of the importance of polyploidy has inspired research highlighting the likelihood that ploidy elevation will often confer major phenotypic (reviewed in Ramsey and Ramsey 2014; Frawley and Orr‐Weaver 2015; *e.g*., Neiman et al. 2009; Balao et al. 2011; Selmecki et al. 2015; Neiman et al. 2016) and genomic (*e.g*., Hollister et al. 2012; Martin and Husband 2012; Yant et al. 2013; Selmecki et al. 2015) consequences. This research has demonstrated that polyploidy can influence many important individual‐ and population‐level traits, ranging from likely positive effects such as increased enzymatic activity (Levin 1983), higher levels of gene expression (De Godoy et al. 2008; Neiman et al. 2009), and greater adaptive potential (Otto and Whitton 2000; Balao et al. 2011; Martin and Husband 2012; Selmecki et al. 2015) to costs associated with ploidy effects on organismal ecology (*e.g*., increased sensitivity to nutrient limitation, Neiman et al. 2013b,c) and genomic mutational load (Otto and Whitton 2000). While this growing body of research highlights the likelihood that ploidy changes are important with respect to organismal ecology and lineage evolution, the overall biological significance of polyploidy remains unclear (Otto and Whitton 2000; Mable et al. 2011; Leslie 2014; Frawley and Orr‐Weaver 2015; Schoenfelder and Fox 2015).

Until recently, polyploidy was thought to be so rare among animals that it was generally inconsequential (Mable 2004; Mable et al. 2011). The frequent presumption that animal polyploids are uncommon, inviable, and/or infertile is likely to explain both why most studies of the consequences of polyploidy have focused on plants and why the phenotypic consequences of polyploidy in animals are so poorly understood (Mable 2004). Here, we use a powerful natural animal system to address whether and to what extent polyploidy influences life history traits like growth rate, body size, and age at reproductive maturity. Life history traits are an appropriate and important focus for the study of the ecological and evolutionary importance of polyploidy because (1) life history traits are often main determinants of fitness (*e.g*., Levy and Feldman 2002; Otto 2007; Ramsey 2011), and (2), ploidy level has the potential to influence life history traits in both positive and negative ways (*e.g*., Cavalier‐Smith 1978; Levin 1983). For example, if polyploids have higher per‐organism gene expression (*e.g*., De Godoy et al. 2008; Neiman et al. 2009), the positive correlation between organismal RNA content and growth rate (Hessen et al. 2010) means that polyploids might be expected to grow more rapidly than diploids (Neiman et al. 2013b,c). Conversely, polyploidy might be associated with relatively slow growth and maturation if increased per‐cell DNA content causes decreased metabolic rate (Cavalier‐Smith 1978; Hessen et al. 2013) and/or increased cell cycle duration (Davies and Rees 1975; Bennett and Leitch 2005; Gregory 2005).

*Potamopyrgus antipodarum*, a New Zealand freshwater snail, is well suited to empirically evaluate connections between ploidy level, reproductive mode, and life history variation because it combines extensive variation in key life history traits (Jacobsen and Forbes 1997; Jokela et al. 1997a, 1999; Jensen et al. 2001; Neiman et al. 2013c; Krist et al. 2014) with widespread within‐ and across‐population ploidy polymorphism (diploids, triploids, and individuals that exceed triploidy; hereafter “tetraploids”; “2x,” “3x,” “4x,” respectively) (Neiman et al. 2011; Paczesniak et al. 2013). Like many other polyploid animals (reviewed in Otto and Whitton 2000; Neiman and Schwander 2011; Neiman et al. 2014), polyploid *P. antipodarum* are obligately asexual, producing nonrecombinant eggs via ameiotic parthenogenesis (Phillips and Lambert 1989). The existence of triploid and tetraploid asexual *P. antipodarum* means that we can use comparisons between asexuals of different ploidy levels as well as between sexuals and asexuals to simultaneously study the consequences of polyploidy and identify costs and benefits associated with sexual versus asexual reproduction, another key unanswered question in evolutionary biology. An additional strength of the *P. antipodarum* system in this context is that, unlike many polyploid animals and plants (Bierzychudek 1985; Otto and Whitton 2000; Neiman and Schwander 2011), triploid and tetraploid *P. antipodarum* are not hybrids. Rather, polyploid *P. antipodarum* are the products of multiple separate ploidy elevation events from lower ploidy *P. antipodarum* (Neiman et al. 2011, 2012; Paczesniak et al. 2013; Soper et al. 2013). The implications are that each different triploid and tetraploid *P. antipodarum* lineage constitutes a distinct natural experiment into the consequences of ploidy elevation and the absence of sex.

Here, we use a common garden approach to evaluate whether the means and variances of growth rate, time to reproductive maturity, and adult female body size differ across ploidy levels and reproductive modes. We chose to focus on these three life history traits because they are all likely to be important determinants of fitness in female *P. antipodarum* (Krist and Lively 1998; Tibbets et al. 2010; McKenzie et al. 2013). A positive association between ploidy level and one or more of these traits would be an exciting discovery, indicating that ploidy elevation confers life history benefits in a natural animal system. This result would also suggest that asexual *P. antipodarum* are likely to realize, at least in some environments, an even greater than the twofold advantage generated by allocating resources to daughters instead of sons (the twofold “cost of males”; Maynard Smith 1978; *e.g.,* Jokela et al. 1997b). Such a result would also emphasize the likelihood that the selective maintenance of diploid sexual *P. antipodarum* is a consequence of direct benefits of sex (*e.g*., recombination). By contrast, decreased growth and/or maturation rate as ploidy level increases would implicate costs of polyploidy as a potential contributor to the relative scarcity of tetraploid versus triploid asexual *P. antipodarum* (*e.g*., sixfold more 3x than 4x *P. antipodarum* identified in a comprehensive study by Neiman et al. 2011; also see Neiman et al. 2013b,c; Paczesniak et al. 2013) and as a potential contributor to the success of sexual *P. antipodarum*. We also used our data to evaluate a fundamental component of many hypotheses for sex as well as a potential evolutionary advantage of ploidy elevation: that sex, via recombination, and ploidy elevation, via the availability of extra allelic copies (Otto and Whitton 2000), will increase the phenotypic variance of heritable traits among sexually produced offspring relative to asexually produced offspring (Barton 1995) and in higher ploidy versus lower ploidy offspring.

## Materials and Methods

### Animals

We initiated the experiment with a total of 169 adult (>3 mm) female *P. antipodarum* (“founding females”) isolated from either field collections or laboratory cultures between January 2011 and May 2011. 140 of the founders, representing 20–40 females collected from each of six New Zealand lakes, were sampled from their native lake in January 2011 (Table 1). With the exception of Alexandrina *Isoetes* snails, all *P. antipodarum* were sampled from shallow locations (<2 m in depth). The Alexandrina *Isoetes* individuals were sampled from depths of ~2–4 m. In order to increase the genetic and geographic diversity of the *P. antipodarum* in the experiment, we also included one founding female from each of 14 triploid and 15 tetraploid asexual laboratory cultures (“lineages”) (characterized in Neiman et al. 2012). These lineages were descended from single females originally collected from eight different New Zealand lakes in January 2009 (Neiman et al. 2011, 2012; Table 1).

**Table 1:** Characteristics of founding females and G1 offspring

Lake of origin	Source	# Founding females	# Founders that reproduced	Males added	# 2x Families	# 3x Families	# 4x Families
Alexandrina (shallow)	Field	20	16	Yes	3	10	0
Alexandrina (*Isoetes*)	Field	20	12	Yes	4	7	0
Clearwater	Laboratory	1	1	No	0	1	0
Clearwater	Laboratory	1	1	No	0	1	0
Grasmere	Field	20	14	Yes	1	12	0
Gunn	Laboratory	1	1	No	0	0	1
Gunn	Laboratory	1	1	No	0	0	1
Gunn	Laboratory	1	1	No	0	0	1
Gunn	Laboratory	1	1	No	0	0	1
Gunn	Laboratory	1	1	No	0	0	1
Gunn	Laboratory	1	1	No	0	1	0
Gunn	Laboratory	1	1	No	0	0	1
Gunn	Laboratory	1	1	No	0	0	1
Gunn	Laboratory	1	1	No	0	0	1
Gunn	Laboratory	1	1	No	0	1	0
Haupiri	Field	20	7	Yes	0	3	1
Heron	Field	20	19	Yes	0	18	1
Kaniere	Laboratory	1	1	No	0	1	0
Kaniere	Laboratory	1	1	No	0	1	0
Okareka	Laboratory	1	1	No	0	1	0
Okareka	Laboratory	1	1	No	0	1	0
Okareka	Laboratory	1	1	No	0	1	0
Okareka	Laboratory	1	1	No	0	1	0
Poerua	Laboratory	1	1	No	0	1	0
Poerua	Laboratory	1	1	No	0	1	0
Poerua	Laboratory	1	1	No	0	0	1
Poerua	Laboratory	1	1	No	0	0	1
Poerua	Laboratory	1	1	No	0	1	0
Poerua	Laboratory	1	1	No	0	1	0
Rotoiti	Laboratory	1	1	No	0	0	1
Rotoiti	Laboratory	1	1	No	0	0	1
Rotoiti	Laboratory	1	1	No	0	1	0
Rotoiti	Laboratory	1	1	No	0	0	1
Rotoiti	Laboratory	1	1	No	0	0	1
Rotoroa	Field	20	10	Yes	1	4	0
Selfe	Field	20	11	Yes	3	5	0
Taylor	Laboratory	1	1	No	0	1	0
Waikaremoana	Laboratory	1	1	No	0	1	0

### Snail housing and care

All founding females were individually housed in a one‐liter cup filled with ~200 mL carbon‐filtered tap water and maintained in a room held at 16°C and under a 16:8 light/dark cycle. Each snail was fed three times per week with dried *Spirulina*, a common laboratory food source for *P. antipodarum* (*e.g*., Neiman et al. 2013a; Zachar and Neiman 2013). All females isolated from the field were housed with males to ensure that sexual females, which are phenotypically indistinguishable from asexual females, would have the opportunity to become fertilized. Because many of these males were field‐collected and thus subject to infection by sterilizing trematode parasites (Winterbourn 1973; Lively 1987), we rotated males through all cups housing field‐collected females on a biweekly basis to ensure that all females had access to fertile males. Because these parasites are sterilizing, any sexual female that did not reproduce (and, hence, that may have been sterilized) was excluded from all subsequent analyses.

### Isolation of offspring

We checked the cup of each founding female under a dissecting microscope three times per week for newly born offspring (G1), recorded the date each G1 was found, and then removed and housed each G1 individually in its own cup. These isolated offspring were housed and maintained in the same manner (including male rotation through cups) as the founding females. This process was repeated for the first eight G1 offspring produced by each founding female. We snap‐froze and stored at −80°C one additional offspring produced by each founding female, to be used later for flow cytometric determination of nuclear DNA content (*i.e*., ploidy; *e.g*., Neiman et al. 2011).

### Measuring growth and reproductive maturity

We checked the G1 cups once per week until the shell of the G1 calcified. At this point, the snail was visible to the naked eye and ~1.0 mm in length. We then used a camera and a dissecting microscope to capture an image of the G1 snail next to a ruler while it was crawling along the bottom of a petri dish. Next, we imported this image into ImageJ and measured shell length from apex to aperture for each snail. We repeated this process weekly until the G1 reached 3.0 mm in shell length, ~0.5–1.5 mm below the shell length at which reproduction typically commences in female *P. antipodarum* (Winterbourn 1970; Tibbets et al. 2010; McKenzie et al. 2013; Fig. 3, present study). At this point, we began rotating males through the cup for G1 snails produced by field‐collected founding females and checking all cups three times a week for offspring (G2). Upon finding a G2 (mean 109.103 ± SD 64.055 days after the 3.0 mm threshold), we recorded the date of birth (accurate within 2–3 days; hereafter, “age at maturity”), and then used a dissecting microscope, camera, and ImageJ software to measure the shell length of the now‐reproductively active G1. *Potamopyrgus antipodarum* growth slows markedly at reproduction, and *P. antipodarum* female fecundity is strongly and positively associated with shell length (Winterbourn 1970; Tibbets et al. 2010; McKenzie et al. 2013), meaning that shell length at first reproduction (hereafter, “final length,” defined as our final length measurement) serves as a meaningful reproductive fitness correlate. Throughout this process we performed weekly mortality checks on all G1 snails and recorded the date of death (accurate within a week) for all dead individuals.

### Flow cytometry

Tissue samples for flow cytometry were prepared following Neiman et al. (2011, 2012). We ran the samples on a Becton Dickinson LSR II flow cytometer. A 20 μL sample of chicken red blood cell standard (Lampire Biological Labs, Pipersville, PA) was prepared in the same manner as the snail tissue and was run at the beginning of each flow cytometry session in order to calibrate the machine so the DAPI‐A peak was centered at 80 FL1 units. We then used the FL1 channel to measure the DAPI fluorescence of each nucleus, which indicates DNA content and thus ploidy level, and used FlowJo software (Version 8.8.7; Tree Star, Inc.) to analyze the flow cytometry data.

### Statistical analyses

We began by addressing the primary question of whether ploidy level affected our three focal life history traits: growth rate (represented as growth (mm) per day until 3.0 mm in shell length) for G1 females, age at maturity for G1 females, and final length for G1 females. We provide definitions for these traits in Table 2. We first used the Kolmogorov–Smirnov (K‐S) test to evaluate whether each of the three dependent variables met the normal distribution requirement of parametric statistical analysis. While final length data were distributed normally (K‐S statistic = 0.041, df = 302, *P *=* *0.200), the growth rate (K‐S statistic = 0.073, df = 302, *P *=* *0.001) and age at maturity data (K‐S statistic = 0.099, df = 302, *P *<* *0.0001) exhibited significant deviations from normality. After a natural log transformation, the age at maturity data (K‐S statistic = 0.040, df = 351, *P *=* *0.200) were distributed normally. We were able to achieve a normal distribution with the growth rate data following a cube root transformation (K‐S = 0.034, df = 365, *P *=* *0.200), allowing us to use parametric analyses for all three life history variables for all subsequent analyses.

**Table 2:** Life history trait definitions

Trait	Definition
Growth rate	Growth rate (per day) of G1 until 3.0 mm in shell length
Age at maturity	The age in days of G1 on the date when the first G2 was observed
Final length	Shell length of G1 on the date that the first G2 was observed

The existence of a size threshold at which *P. antipodarum* females first start reproducing (Winterbourn 1970; McKenzie et al. 2013; present study) suggests that variation in growth rate might be associated with variation in age and size at reproductive maturity. With this logic in mind, we used linear regression analyses to evaluate whether we would need to correct for effects of growth rate on age at maturity and final size, respectively.

Growth rate was significantly associated with both age at maturity (linear regression; beta = −0.652, *F *=* *237.130, *P *<* *0.0001) and final length (linear regression; beta = 0.221, *F *=* *15.488, *P *<* *0.0001), so we saved the residuals from these regression analyses as growth rate‐corrected estimates of age at maturity and final length. We then used these residuals (along with growth rate itself) as dependent variables in general linear model analyses evaluating whether the fixed factor of ploidy level influenced the dependent variables of growth rate, age at maturity, and final length. We controlled for descent from the same founding female by nesting the random factor of family (defined as all G1 *P. antipodarum* produced by the same founding female) within ploidy level, and used post hoc Tukey's honestly significant difference analyses to determine whether there were differences in growth rate, age at maturity, and/or final length between particular ploidy levels. Because shell length in female *P. antipodarum* is positively associated with fecundity (Winterbourn 1970; McKenzie et al. 2013), we also conducted these same analyses with the raw (*i.e*., non‐growth rate‐corrected) final length data. While the outcome of these analyses could be driven by the positive association between growth rate and final length, they also provide us with a straightforward means of assessing whether shell length and thus fecundity differ across ploidy levels and/or reproductive modes. By similar logic, because age at maturity (regardless of associations with growth rate) is likely to be an important determinant of reproductive success in *P. antipodarum*, which is subject to both parasitic sterilization (Jokela and Lively 1995) and juvenile‐stage predation (Levri 1998), we also performed a set of analyses using the raw age at maturity data.

Because our regression analyses suggested that variation in growth rate might be an important determinant of age at maturity and final length, we used correlation analyses to evaluate whether and how growth rate was correlated with the other two variables. In order to account for nonindependence of family members, we first calculated mean family values for each of the three life history variables from the individual‐level data. We then used the K‐S test to evaluate whether these family means were normally distributed. None of the datasets exhibited significant deviations from normality (growth rate: K‐S statistic = 0.056, df = 95, *P *=* *0.200; age at maturity: K‐S statistic = 0.059, df = 95, *P *=* *0.200; final length: K‐S statistic = 0.065, df = 95, *P *=* *0.200), so we used the parametric Pearson's r approach for both correlation analyses.

The maintenance of intraspecific life history variation is often linked to spatially variable selection (recently reviewed in Richardson et al. 2014), and the existence of substantial genetic variation for all three life history traits within *P. antipodarum* (present study; also see Jokela et al. 1997a; Jacobsen and Forbes 1997; Jensen et al. 2001; Neiman et al. 2013c) inspired us to evaluate potential effects of lake of origin. These lake‐level analyses are particularly interesting in light of evidence that the strength and type of selection for life history traits such as growth rate might vary across or within *P. antipodarum* populations (Jokela and Lively 1995; Krist et al. 2014). We evaluated our dataset for lake effects using general linear models to determine whether the fixed factors of lake of origin and random factor of family (~genetic variation; nested within lake of origin) influenced growth rate, age at maturity, and final length. The sample sizes for diploid and tetraploid families were too small (four lakes with 2x families, five lakes with 4x families) for meaningful analysis, so we confined these analyses to the 76 triploid families from 14 lakes.

We addressed the question of whether sexual reproduction confers higher variance in fitness‐related traits relative to asexuals by comparing the variance for each of the three life history traits that we measured in sexual and asexual *P. antipodarum* (following Becks and Agrawal 2011). We began by using a variance components analysis to estimate the variance of each trait for each of the 12 sexual and 93 asexual families that we included in our experiment. K‐S analyses revealed that all three sets of family variances required transformation to meet the normal distribution requirement of parametric statistical analysis (final length: K‐S statistic = 0.171, df = 83, *P *<* *0.0001; growth rate: K‐S statistic = 0.157, df = 79, *P *= <0.0001; age at maturity: K‐S statistic = 0.223, df = 85, *P *<* *0.0001). After natural log transformations of these data, the variances of age at maturity (K‐S statistic = 0.087, df = 77, *P *=* *0.200) and final length (K‐S statistic = 0.094, df = 77, *P *=* *0.091) were normally distributed. The growth rate variance data met the normality requirement following a cube root transformation (K‐S statistic = 0.072, df = 79, *P *=* *0.200).

We then used these transformed family variances as the dependent variables in univariate general linear models with reproductive mode as a fixed factor, and estimated 95% confidence intervals around the mean trait variance (calculated by taking the mean of the variances for sexual and asexual families, respectively) for sexual versus asexual families with 1000 bootstrap replications. We used the same procedure to compare the mean family variances in the 76 asexual triploid families versus the 17 tetraploid families (with ploidy as a fixed factor). The ploidy analysis allowed us to address whether ploidy level per se might affect the variance in expression of life history traits. All statistical analyses (including power analyses, using the “opower” function) were conducted with IBM SPSS Statistics version 21.

## Results

### Effects of ploidy and reproductive mode on life history traits

#### Growth rate until 3 mm

**Figure 1:**
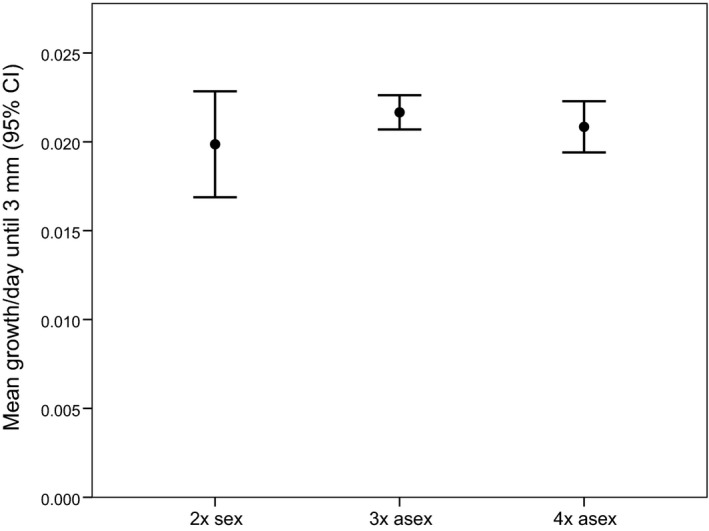
Mean growth rate (mm/day) until 3.0 mm in shell length across ploidy levels. We show untransformed data here in order to facilitate interpretability; the comparisons involving the cube root‐transformed data are qualitatively identical (Fig. S1).

We did not detect a significant effect of ploidy level on growth rate (Figs 1 and S1, Table 3; Tukey's honestly significant difference: 2x–3x: *P *=* *0.441; 2x–4x: *P *=* *0.064; 3x–4x: *P *=* *0.140). Because growth rate in triploids and tetraploids was statistically indistinguishable, we combined the triploids and tetraploids into one “asexual” category and then conducted a general linear model analysis identical to that used for the ploidy analysis except that we replaced the fixed factor of ploidy with the fixed factor of mode of reproduction (sexual vs. asexual), allowing us to address whether reproductive mode might influence growth rate in a manner only detectable when all asexuals were pooled. This analysis, however, did not provide any evidence for a reproductive mode effect on growth rate (Table 3).

**Table 3:** Summary of outcomes of univariate general linear models evaluating the effect of ploidy level and reproductive mode on life history traits. We pooled triploids and tetraploids for “reproductive mode” analyses contrasting the diploid sexuals with the triploid and tetraploid asexuals only if post hoc Tukey's tests conducted as part of “ploidy” analyses showed that triploids and tetraploids were not statistically distinguishable (*P *>* *0.05)

Trait	Effect	*F*(df)	*P*
Growth rate	Ploidy	1.347 (2, 129.52)	0.264
	Family (ploidy)	2.038 (99, 263.00)	<0.0001
	Reproductive mode	0.305 (1, 164.06)	0.581
	Family (reproductive mode)	2.082 (99, 264.00)	<0.0001
Age at maturity	Ploidy	2.182 (2, 168.99)	0.116
	Family (ploidy)	1.383 (97, 222.00)	0.026
	Reproductive mode	3.850 (1, 245.68)	0.055
	Family (reproductive mode)	1.377 (98, 222.00)	0.027
	Ploidy^a^	4.313 (2, 139.40)	0.015
	Family (ploidy)^a^	2.644 (99, 280.00)	<0.0001
	Reproductive mode^a^	6.940 (1, 177.00)	0.009
	Family (reproductive mode)^a^	2.670 (100, 280.00)	<0.0001
Final length	Ploidy	2.604 (2, 143.27)	0.077
	Family (ploidy)	1.774 (93, 208.00)	<0.0001
	Ploidy^a^	0.067 (2, 109.13)	0.510
	Family (ploidy)^a^	4.647 (94, 260.00)	<0.0001
	Reproductive mode^a^	0.956 (1, 134.62)	0.330
	Family (reproductive mode)^a^	4.647 (94, 260.00)	<0.0001

a Denotes data uncorrected for significant associations with growth rate.

#### Age at maturity

While there was not a significant main effect of ploidy level on age at maturity (growth rate‐corrected) (Table 3), Tukey's post hoc pairwise analyses revealed that diploids matured significantly more slowly than triploids (*P *=* *0.023) but were not statistically distinguishable from tetraploids (*P *=* *0.145). Triploid and tetraploid asexuals were also statistically indistinguishable (*P *=* *0.636) (Fig. S2). We thus combined triploids and tetraploids into an asexual category and used the same general linear model approach to evaluate whether sexuals and asexuals differed in age to maturity, allowing us to address whether reproductive mode (rather than ploidy per se) influenced age at maturity. This analysis revealed a marginally significant effect of reproductive mode (*P *=* *0.055).

**Figure 2:**
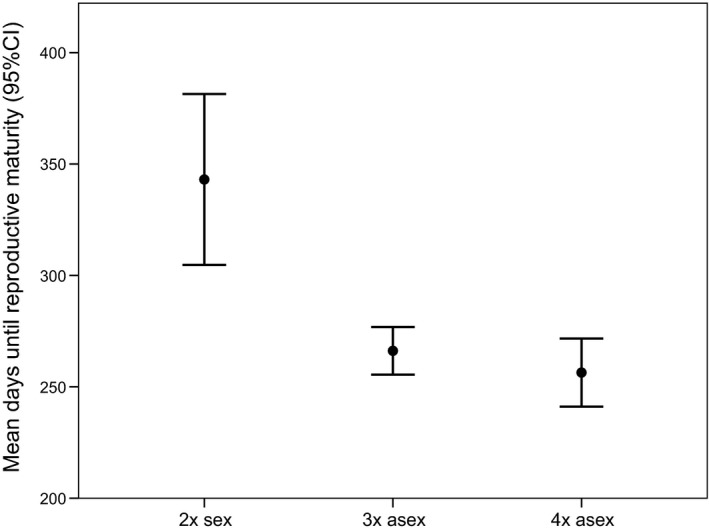
Mean days until reproductive maturity across ploidy levels. We show uncorrected and untransformed data here in order to facilitate interpretability; the comparisons involving the natural log‐transformed residual data are qualitatively similar, although the removal of the effect of growth rate rendered the overall effect of reproductive mode and the diploid–tetraploid comparisons nonsignificant (Fig. S2).

The growth rate‐uncorrected age at maturity analyses did reveal a significant effect of ploidy level (Table 3), with diploids maturing at a significantly later age than both triploids and tetraploids (Tukey's honestly significant difference, *P *<* *0.0001; Fig. 2). Because there was no significant difference in age at maturity between triploids and tetraploids (*P *=* *0.101), we again combined the triploids and tetraploids into an asexual category and used the same univariate general linear model approach as before to address the effect of reproductive mode on uncorrected age at maturity. Here, we detected a significant effect of reproductive mode (Table 3) on age to maturity, driven by the ~40% increase in the number of days it took sexuals to achieve reproductive maturity relative to asexuals (Fig. 2). The difference in outcome between the reproductive mode analyses for the growth rate‐corrected versus uncorrected age at maturity data suggests that the lower rate of maturation in the sexuals is ultimately driven by growth rate effects (also see Figs 4 and S4).

#### Final length

Although there was no significant main effect of ploidy level on final length (growth rate residuals) (*P *=* *0.077; Table 3, Fig. S3), tetraploids were significantly larger than triploids (*P *=* *0.026). Diploids and triploids (*P *=* *0.791) and diploids and tetraploids (*P *=* *0.099) were statistically indistinguishable.

**Figure 3:**
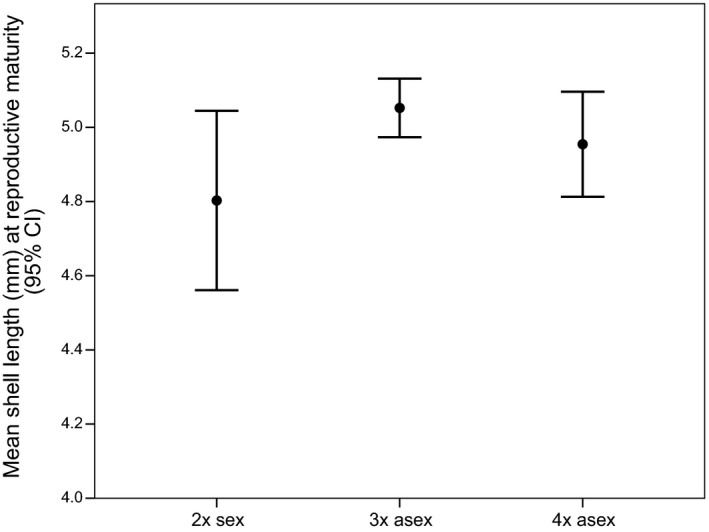
Mean shell length at reproductive maturity across ploidy levels. We show uncorrected and untransformed data here in order to facilitate interpretability; the comparisons involving the natural log‐transformed residual data are similar in that there is no significant main effect of ploidy. These comparisons differ in that triploids are significantly longer than diploids in the uncorrected and untransformed dataset and that tetraploids are significantly longer than the triploids in the transformed residual dataset (Fig. S3).

Analysis outcomes using the uncorrected final length data (Table 3) showed that diploid sexual female *P. antipodarum* (mean = 4.803 ± 0.611 mm) had significantly shorter shell lengths at reproductive maturity than their triploid counterparts (triploid mean = 5.037 ± 0.625 mm, *P *=* *0.033; Fig. 3). There was not a significant difference in shell length between triploids and tetraploids (tetraploid mean = 4.903 ± 0.715 mm; *P *=* *0.073), indicating that the significant differences in growth rate‐corrected final length that we observed between triploids and tetraploids are not evident when the growth rate–shell length association is left intact. Diploids and tetraploids (*P *=* *0.596) were also statistically indistinguishable (Fig. 3).

The intermediate ploidy level (triploid) *P. antipodarum* had the longest shells, suggesting that ploidy level variation per se does not account for the relatively short shell length of diploid sexual females. A reproductive mode‐focused univariate general linear model analysis comparing the final length of diploid sexuals to the pooled triploid and tetraploid asexuals showed no significant difference in final length between sexuals and asexuals (Table 3; also see Fig. 3).

#### Growth rate from 3 mm until reproductive maturity

**Figure 4:**
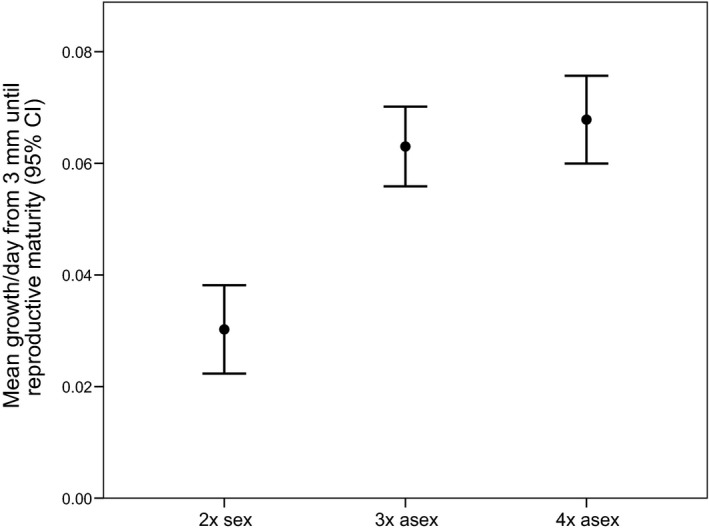
Mean growth rate (mm/day) from 3.0 mm in length until reproductive maturity. We show uncorrected and untransformed data here in order to facilitate interpretability; the comparisons involving the natural log‐transformed residual data are qualitatively identical.

The fact that female *P. antipodarum* of all three ploidy levels and both reproductive modes usually do not reproduce until reaching at least 4.0 mm in shell length combined with the markedly slower attainment of reproductive maturity in sexual versus asexual females and the strong negative correlation between growth rate and age at maturity suggests that a potential driver of these differences in rate of maturation is relatively rapid growth in asexual versus sexual *P. antipodarum*. While these differences were not apparent in our comparisons of growth rate to 3.0 mm (Fig. 1), a general linear model evaluating how the fixed factor of reproductive mode influenced the dependent variable of days between the attainment of 3.0 mm in shell length and the date of first reproduction (with the random factor of family nested within reproductive mode) indicated that asexual *P. antipodarum* take significantly fewer days (56% of days relative to sexuals) to reproduce after reaching 3.0 mm than their sexual counterparts (*F*_(1, 190.348)_ = 15.271, *P *<* *0.0001; Figs 4, S4). This result is consistent with a scenario where asexual *P. antipodarum* reproduce at an earlier age than sexual *P. antipodarum* because the former grow more rapidly than the latter. The significantly higher age at maturity of sexual versus asexual *P. antipodarum* revealed by the general linear model analysis using uncorrected (i.e*.,* growth rate association not removed) age at maturity data compared to the only marginally significant outcome of the same analysis using growth rate‐corrected age at maturity data further supports this conclusion Table 3, Figs 2, S2.

Another line of support for the possibility that higher growth rate underlies the relatively early reproductive maturity of asexual *P. antipodarum* comes from a Pearson's correlation analysis revealing a significant negative correlation between growth rate and age at maturity (*R* ^2^ = −0.622, *P *<* *0.0001) but no relationship between growth rate and final size (*R* ^2^ = 0.098, *P *=* *0.098). The tight association between growth rate and age at maturity highlights the likelihood that growth rate is an important fitness‐related trait in *P. antipodarum*.

### Genetic variation for all life history traits

We found a significant effect of family in both the ploidy level and reproductive mode analyses for all life history traits (Table 3). These results indicate the existence of substantial genetic variation for growth rate, age at maturity, and final length in natural populations of sexual and asexual *P. antipodarum*.

### Evaluating effects of lake of origin on life history variation in asexual triploid *P. antipodarum*

We did not detect significant effects of lake of origin on any of our three focal life history traits (growth rate: *F*_(13, 61.597)_ = 0.973, *P *=* *0.488; growth rate‐corrected age at maturity: *F*_(13, 56.101)_ = 0.692, *P *=* *0.763; growth rate‐corrected final size: *F*_(13, 61.460)_ = 1.691, *P *=* *0.086). The power of these analyses was moderately high (0.522–0.818), suggesting that we would have detected strong effects of lake of origin. There was a significant effect of family, and thus genetic variation within lakes, for growth rate (*F*_(62, 184)_ = 1.728, *P *=* *0.003) and final size (*F*_(57, 185)_ = 1.414, *P *<* *0.0001), but not for age at maturity (*F*_(60, 164)_ = 1.254, *P *=* *0.133), indicating low levels of genetic variation for age at maturity (after the effect of growth rate is removed) relative to the other two traits. The analysis of the uncorrected age at maturity data still detected no effect of lake (*F*_(13, 62.141)_ = 1.582, *P *=* *0.115, observed power = 0.786) but did reveal a significant effect of family (*F*_(61, 208)_ = 2.378, *P *<* *0.0001). This latter result suggests that while age at maturity is heritable, selection on this trait will likely impose correlated selection on growth rate, with the implication that these two traits cannot be selected independently.

### Effects of reproductive mode and ploidy level on life history trait variances

There were no significant differences in mean variance in any of the three life history traits between reproductive modes or between triploid and tetraploid asexuals (Table 4). These results suggest that sexual reproduction may not create a variance‐related benefit of sex in *P. antipodarum*, at least for these traits and in a laboratory environment, although the low statistical power (< 0.384) of these analyses means that this conclusion should be viewed as preliminary. The absence of obvious differences in variance between triploids and tetraploids also indicates that phenomena associated with extra genomes (*i.e*., higher heterozygosity, mitotic instability) do not necessarily influence offspring trait expression.

**Table 4:** The outcome of univariate general linear models comparing family variances in growth rate, age at maturity, and final length between sexual (Sex) and asexual (Asex) *P. antipodarum* and triploid and tetraploid asexual *P. antipodarum*. ▵ = Variance_Sex_ − Variance_Asex_ or Variance_Triploid_−Variance_Tetraploid_

	Mean Variance^a^	95% CI	Δ	95% CI for Δ	*P*‐value for Δ
Growth rate					
Sex	0.022	0.015 to 0.030	−0.005	−0.003 to 0.013	0.247
Asex	0.027	0.024 to 0.029			
Triploid	0.021	0.017 to 0.025	0.001	−0.007 to 0.007	0.894
Tetraploid	0.020	0.015 to 0.026			
Age at maturity					
Sex	8.048	6.727 to 9.228	0.341	−1.550 to 1.069	0.613
Asex	7.707	7.390 to 8.080			
Triploid	7.849	7.452 to 8.239	0.695	−1.577 to 0.106	0.118
Tetraploid	7.154	6.261 to 7.832			
Final length					
Sex	−2.240	−3.042 to −1.439	−0.192	−1.081 to 1.466	0.759
Asex	−2.048	−2.290 to −1.810			
Triploid	−2.060	−2.345 to −1.780	0.019	−0.647 to 0.601	0.963
Tetraploid	−2.079	−2.679 to −1.513			

a Values are calculated from the transformed variances.

## Discussion

We used a common garden approach to evaluate whether life history traits differ across ploidy level and reproductive mode in *Potamopyrgus antipodarum*, an important natural model system for the study of ploidy variation, the evolution of sex, host–parasite coevolution, and invasion biology. Our study revealed extensive genetic variation for all three life history traits that we measured (growth rate, age at maturity, final length) and suggested that reproductive mode was likely to be a more important contributor to life history trait expression than ploidy level. In particular, we found that diploid sexual *P. antipodarum* grow and mature at a markedly lower rate than their polyploid asexual counterparts. The consistent absence of detectable differences in life history trait means or variances between triploid and tetraploid asexual *P. antipodarum*, with the exception of growth rate‐corrected final length, indicates that effects of ploidy level are not likely to explain the significantly longer time to maturity in sexual diploid *P. antipodarum*.

Our results generally depart from other studies that have evaluated the life history consequences of ploidy elevation, which often find that polyploids develop more slowly (*e.g*., Lowcock 1994; von Well and Fossey 1998; Eliášová and Münzbergová 2014) and have larger bodies (Otto and Whitton 2000; Gregory and Mable 2005) than their diploid counterparts. Nearly all of these previous studies were different from ours in focusing on hybrid and/or plant (usually hybrid) polyploids (Mable et al. 2011), which provides a plausible potential explanation for the striking differences between our study outcomes. We also performed the first comparison of which we are aware of phenotypic variances across lineages featuring natural variation in ploidy level, with no evidence for an increase in trait variance associated with ploidy elevation. Taken together, our results thus suggest that nonhybrid polyploid animals might experience different (and relatively minor) consequences of ploidy elevation relative to their hybrid and/or plant counterparts. This suggestion finds indirect support from the multiple studies (nearly all in plants) that have demonstrated that allopolyploidy has a much larger effect on gene expression than autopolyploidy, implicating hybridization rather than ploidy elevation per se as the causal factor in gene expression changes following a transition to polyploidy (reviewed in Neiman et al. 2013b).

Our results instead suggest that reproductive mode might directly influence life history trait expression, although we cannot formally rule out the alternative explanation that the transition from diploid to triploid confers substantially more consequences than the triploid to tetraploid transition. An important potential role of growth rate as a primary driver of the life history differences that we detected between sexual and asexual *P. antipodarum* is revealed by the combination of an apparent threshold size for female reproductive maturity (~4.0 mm in shell length; also see Winterbourn 1970; McKenzie et al. 2013), the negative association between growth rate and age at reproduction, and the significantly longer time to reproductive maturity of sexual diploid female *P. antipodarum*.

Because a higher growth rate likely translates into fitness advantages (earlier reproduction) for female *P. antipodarum* (also see Tibbets et al. 2010), our results raise the questions of why there exists substantial genetic variation for this trait in *P. antipodarum* and why sexual *P. antipodarum* grow and mature relatively slowly. With respect to the maintenance of genetic variation for growth rate, the well‐documented dependence of growth rate in *P. antipodarum* on environmental variables like food quality (Neiman et al. 2013c; Krist et al. 2014), food quantity (Neiman et al. 2013a), temperature (Dybdahl and Kane 2005), and population density (Neiman et al. 2013a; Zachar and Neiman 2013) suggest that at least some of the genetic variation for growth rate observed in benign laboratory conditions may be suppressed or expressed differently in the more heterogeneous natural populations. Indirect support for this possibility is provided by Neiman et al. (2013c), who found that the growth rate advantages experienced by tetraploid versus triploid *P. antipodarum* when fed a high‐phosphorus diet (notably, a diet higher in phosphorus than the snails in the present study received) disappear under relatively low‐P conditions. The results of Neiman et al. (2013c) emphasize the possibility that we may have detected life history disadvantages associated with polyploidy had we raised the snails in this experiment in harsher (*e.g*., low food quantity or quality) conditions. More broadly, rigorous evaluation of the extent to which environmental variation might influence growth rate and other important life history traits in natural *P. antipodarum* populations will ultimately require quantification of these traits in a wider set of environmental conditions as well as in the field.

Another possibility is that more rapid growth and/or earlier maturation confer other, as yet unmeasured, costs (Blankenhorn 2000; Mangel and Stamps 2001). Such tradeoffs have often been documented in other taxa, and include costs such as an increased rate of developmental deformities (Sibly and Calow 1986), decreased maintenance and repair of proteins and DNA (Roff 1984), and reduced immune (Arendt 1997) and cellular functions (Ricklefs et al. 1998). Under the assumption that these or other costs associated with more rapid growth and maturation might plausibly affect survivorship, we used a Fisher's exact test to compare the proportion of sexual versus asexual and diploid versus triploid versus tetraploid *P. antipodarum* that survived to reproductive maturity. We found that the sexuals were ~17% more likely to die prior to reproduction than the asexuals as a whole (*P *=* *0.0028; Fig. S5). Comparisons of mortality between the diploid sexuals and the triploid (*P *=* *0.0034) and tetraploid (*P *=* *0.0110) asexuals were qualitatively identical (Fig. S6). By contrast, there was no detectable difference in the proportion of triploid versus tetraploid asexuals that died prior to reproduction (*P *=* *1.000). These analyses thus provide no evidence either for a growth rate–mortality tradeoff or obvious effects of elevated ploidy on mortality in our experiment. Altogether, these results support the conclusion that sexual *P. antipodarum* appear to suffer life history disadvantages, with the caveat that because we did not measure lifetime reproductive output in these iteroparous snails (which would require another 2–3 years of study per snail), we cannot rule out the possibility that tradeoffs might be evident at this much longer timescale.

Life history trait variation can also generate population‐level effects that can influence the maintenance of individual‐level variation for these traits (Pfister 1998; Beckerman et al. 2002). Potential connections between individual life history trait expression and population dynamics have already been illustrated in *P. antipodarum* by Pedersen et al. (2009), who showed that a change in fecundity (itself positively associated with shell length in *P. antipodarum*) will have much less of an impact on population growth than proportional changes in other life history traits such as individual growth rate, time to reproductive maturity, or survivorship. The results reported by Pedersen et al. (2009) suggest that feedbacks between individual trait values and population dynamics in *P. antipodarum* are thus more likely to help explain the maintenance of genetic variation for shell length but are less relevant to understanding the maintenance of genetic variation for growth rate and age at maturity.

Another potential non‐mutually exclusive explanation for why sexual *P. antipodarum* grow and mature more slowly than their asexual counterparts is provided by the higher per‐unit mass RNA content (Neiman et al. 2009) and tissue regeneration rate (Krois et al. 2013) of asexual versus sexual *P. antipodarum*. Together, these results hint that asexual *P. antipodarum* may realize tissue‐ and individual‐level growth advantages connected to higher per‐organism gene expression levels (Neiman et al. 2013b). The extent to which this type of mechanism might help explain the life history differences between sexual and asexual *P. antipodarum* will require in‐depth characterization of gene expression levels in sexuals and asexuals and evaluation of how gene expression differences (if any) translate into differences in life history trait expression.

Selection can act very efficiently to promote high‐quality genotypes when asexual populations harbor high genetic diversity (reviewed in Neiman and Linksvayer 2006). This connection between the efficacy of selection and asexual diversity combined with the fact that most asexual *P. antipodarum* have a unique multilocus genotype (Paczesniak et al. 2013; also see Fox et al. 1996) thus raises the possibility of yet another non‐mutually exclusive explanation for the higher performance of asexual *P. antipodarum*: Asexual *P. antipodarum* might grow more rapidly and reproduce earlier than their sexual counterparts because genotypes that contribute to rapid growth and earlier reproduction are not broken up by recombination, allowing for effective selection between different asexual genotypes. Some testable predictions stem from this hypothesis, including the expectations that growth‐related traits in *P. antipodarum* should be affected by multiple loci that are not physically linked (Koehn et al. 1988) and that there should be less variation in life history traits within asexual sibling groups than in sexual sibling groups.

The latter prediction was not upheld in our comparisons of mean trait variance between sexual and asexual families, although the interpretation of this negative result is complicated by the fact that sexual and asexual *P. antipodarum* are sometimes (but not always) genetically distinct (Neiman and Lively 2004; Paczesniak et al. 2013) and the possibility that the offspring produced in our experiment by a single sexual female did not have the same father (also see Soper et al. 2012). Multiple paternity would tend to result in a bias toward inflated variance in sexual families, however, indicating that this particular factor is not likely to explain the absence of significant differences in trait variation in sexually versus asexually reproduced *P. antipodarum*. More broadly, the absence of evidence for increased phenotypic variance in sexual versus asexual *P. antipodarum* families is of direct relevance toward evaluating the set of hypotheses for the maintenance of sexual reproduction that posit benefits of sex associated with the ability to produce phenotypically variable offspring.

Altogether, our survey of life history variation across ploidy levels and reproductive modes in *P. antipodarum* indicates that higher ploidy does not confer obvious life history costs, at least with respect to the traits that we measured. These results suggest that the effects of ploidy elevation, if any, are either weak or nonlinear (*e.g*., the transition from diploid to triploid affects phenotype substantially more than the transition from triploid to tetraploid) under the benign laboratory conditions used in this experiment. In the absence of direct evidence for individual‐ or population‐level costs or tradeoffs associated with more rapid growth or reproduction, the notably slower rate of maturation of sexual versus asexual *P. antipodarum* indicates that the sexuals experience even more than the twofold cost of sex already documented for sexual *P. antipodarum* (Jokela et al. 1997b). Our study serves as a qualitative advance relative to earlier work on *P. antipodarum* by including a focus on effects of both ploidy and reproductive mode, the inclusion of multiple natural lake populations, and the evaluation of both the means and variances of several distinct life history traits. These results are thus likely to extend to the species as a whole, indicating that there exist major benefits associated with sexual reproduction that allow sexual diploid *P. antipodarum* to overcome what appear to be substantial life history disadvantages and persist in some natural populations. Evidence that parasite‐mediated negative frequency‐dependent selection is likely to help favor sexual *P. antipodarum* in at least some New Zealand populations (*e.g*., Lively 1987) is consistent with this conclusion.

## Conflict of Interest

None declared.

## Supporting information


**Figure S1.** Mean growth rate until 3.0 mm in shell length across ploidy levels using the transformed data. We rescaled the *y*‐axis in order to facilitate visual comparisons.

**Figure S2.** Mean days until reproductive maturity across ploidy levels using the growth rate‐corrected transformed data.

**Figure S3.** Mean shell length at reproductive maturity using the growth rate‐corrected transformed data.

**Figure S4.** Mean growth per day from 3.0 mm in length until reproductive maturity using the transformed data. We rescaled the *y*‐axis in order to facilitate visual comparisons.

**Figure S5.** Proportion of sexual and asexuals that reproduced and the proportion of sexuals and asexuals that died prior to reproduction. A Fisher's exact test revealed that a significantly higher proportion of sexuals died prior to reproduction than asexuals (*P = *0.0028).

**Figure S6.** Proportion of 2x, 3x, and 4x snails that reproduced and the proportion of 2x, 3x, and 4x snails that died prior to reproduction. Fisher's exact tests revealed that a significantly higher proportion of sexuals died prior to reproduction than triploid asexuals (*P *=* *0.0034) and relative to tetraploid asexuals (*P *=* *0.0110). There was no significant difference in the proportion of 3x vs. 4x snails that died prior to reproduction (*P *=* *1.0000).
