# Supplementary figures and images for: Effects of polyploidy and reproductive mode on life history trait expression
Source: Ecol Evol. 2016 Jan 11;6(3):765–78. doi: 10.1002/ece3.1934 (PMC4739562; doi:10.1002/ece3.1934)

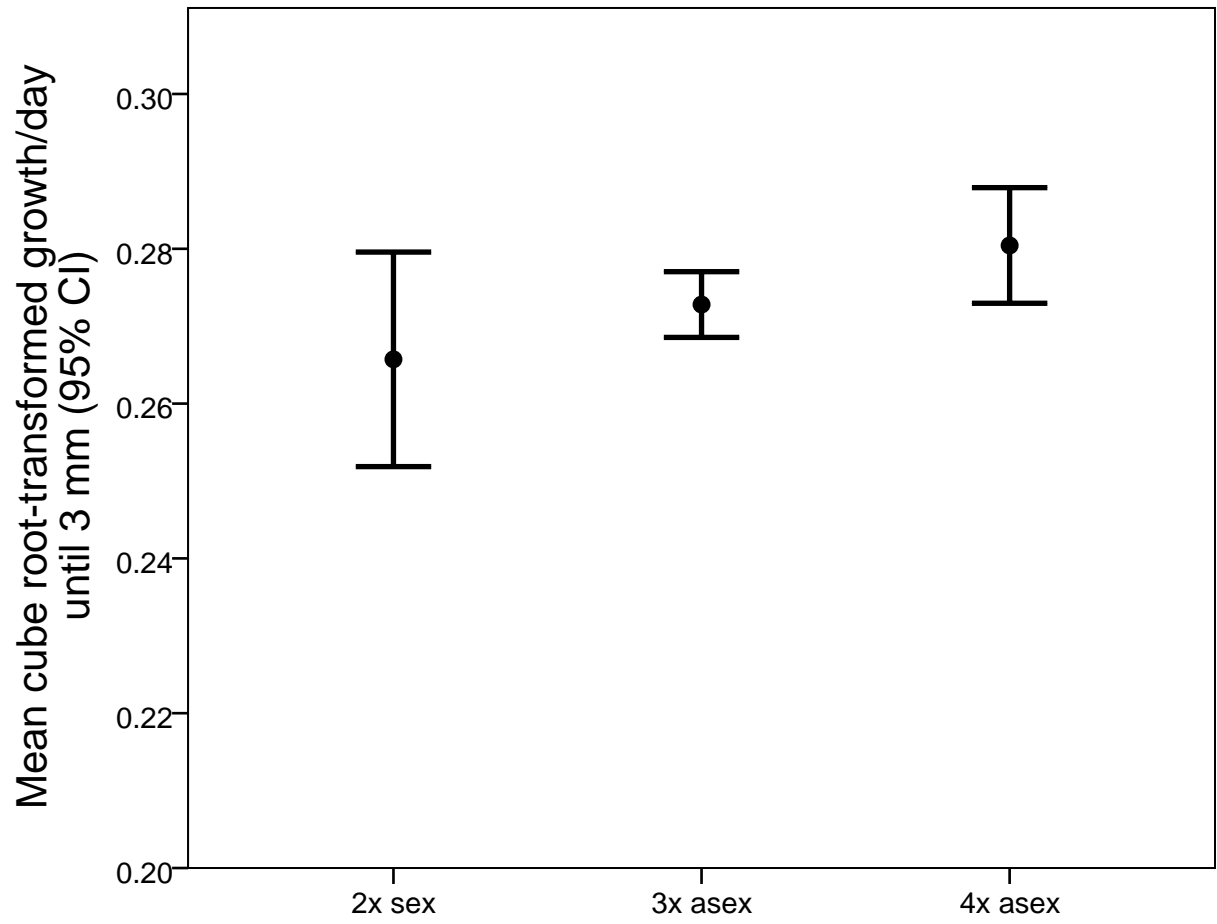

Supplement: Supplementary file 1 — Figure S1. Mean growth rate until 3.0 mm in shell length across ploidy levels using the transformed data. We rescaled the y‐axis in order to facilitate visual comparisons. [file ECE3-6-765-s001.pdf]

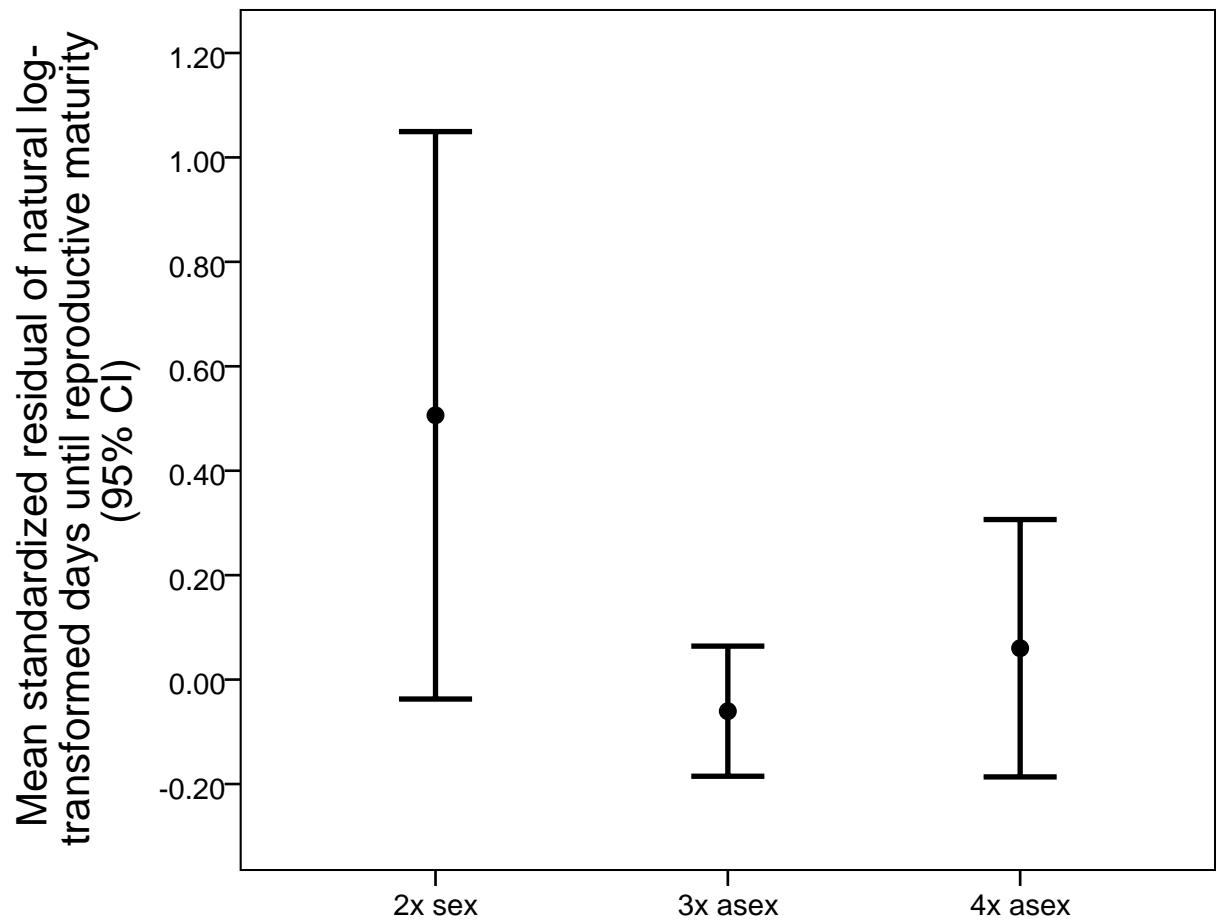

Supplement: Supplementary file 2 — Figure S2. Mean days until reproductive maturity across ploidy levels using the growth rate‐corrected transformed data. [file ECE3-6-765-s002.pdf]

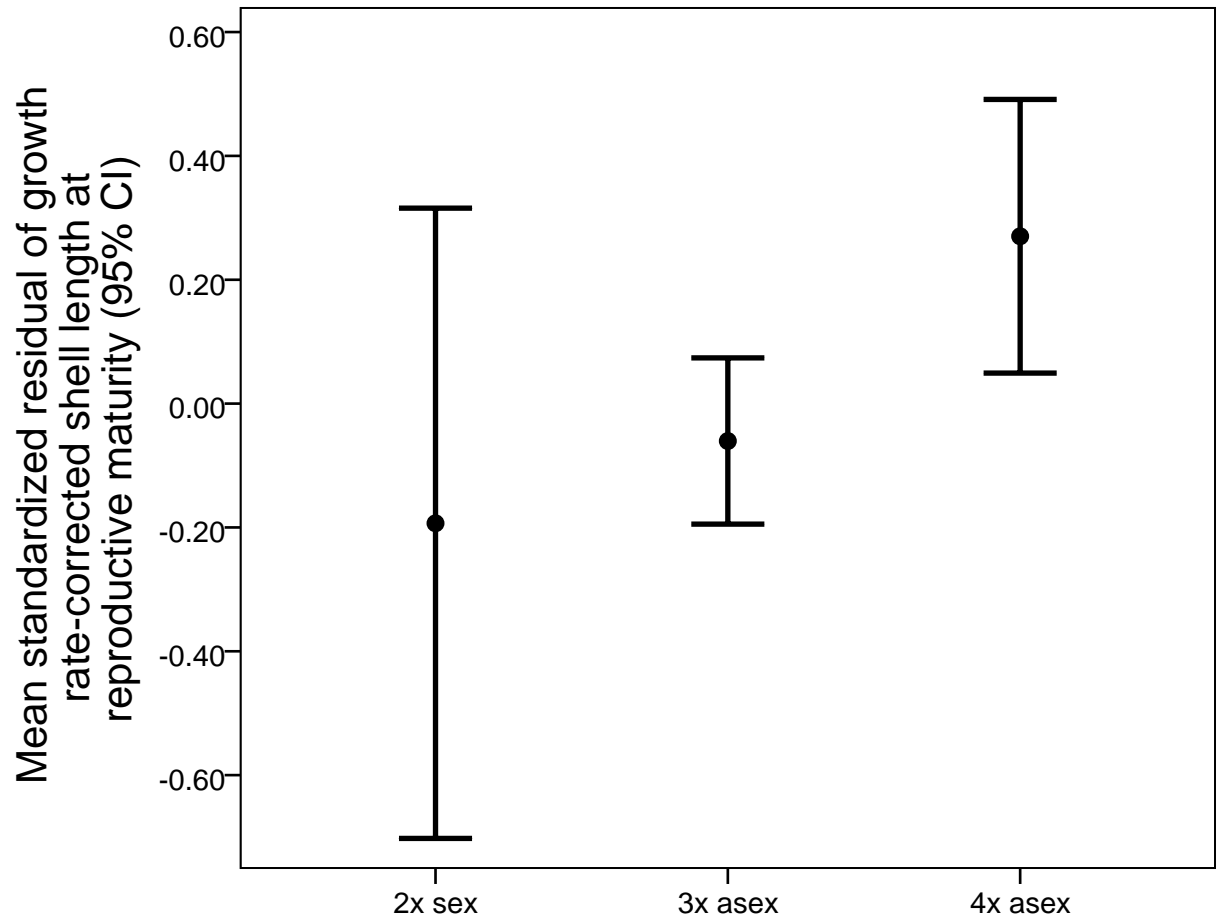

Supplement: Supplementary file 3 — Figure S3. Mean shell length at reproductive maturity using the growth rate‐corrected transformed data. [file ECE3-6-765-s003.pdf]

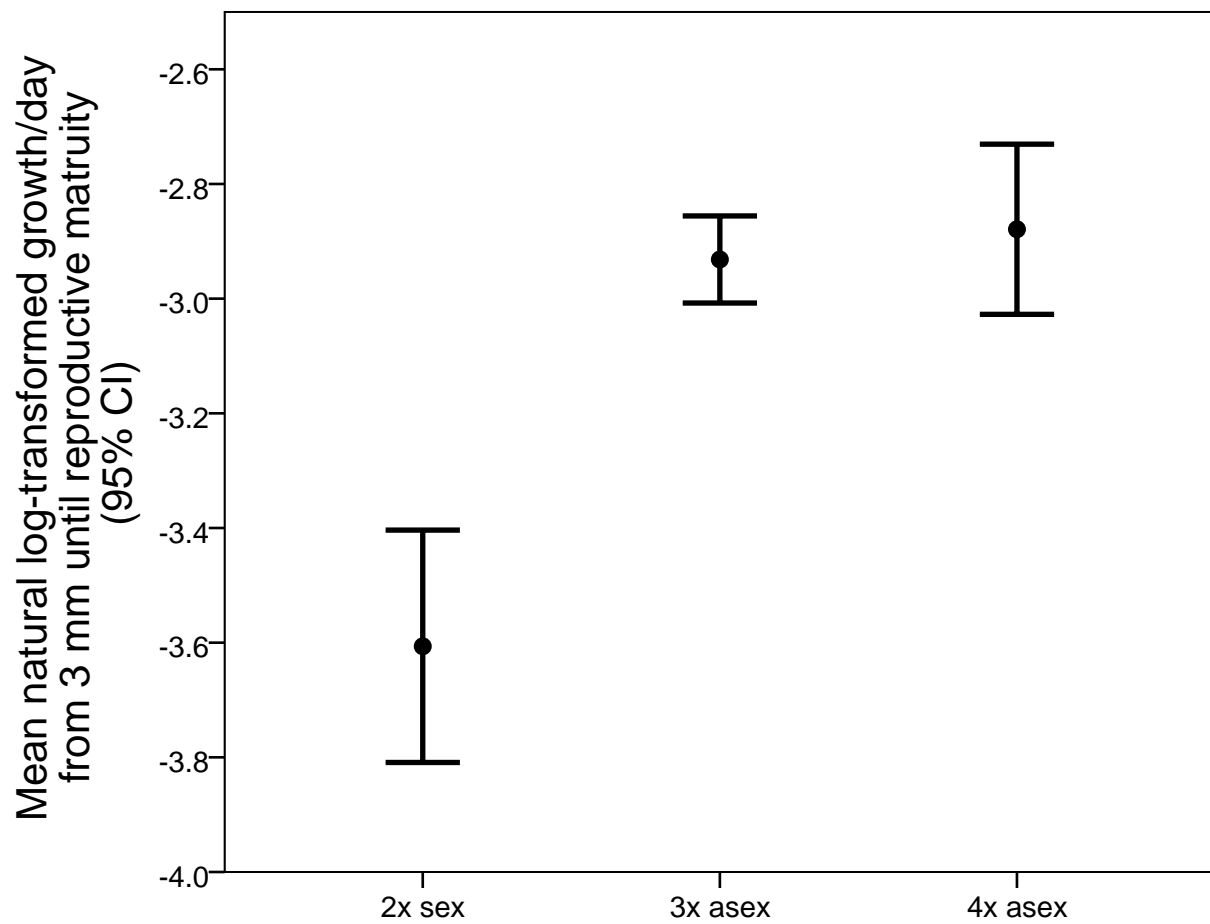

Supplement: Supplementary file 4 — Figure S4. Mean growth per day from 3.0 mm in length until reproductive maturity using the transformed data. We rescaled the y‐axis in order to facilitate visual comparisons. [file ECE3-6-765-s004.pdf]

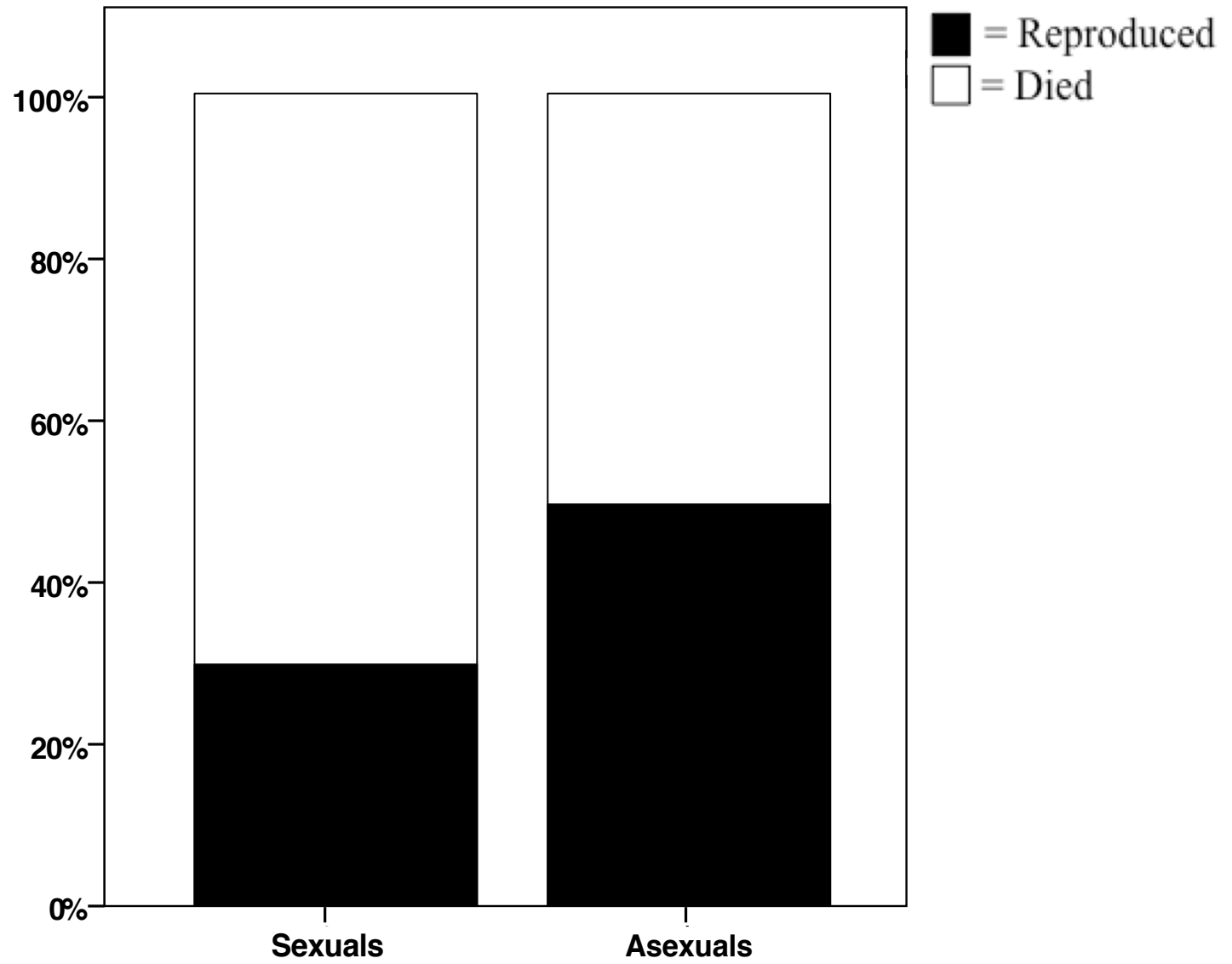

Supplement: Supplementary file 5 — Figure S5. Proportion of sexual and asexuals that reproduced and the proportion of sexuals and asexuals that died prior to reproduction. A Fisher's exact test revealed that a significantly higher proportion of sexuals died prior to reproduction than asexuals (P = 0.0028). [file ECE3-6-765-s005.pdf]

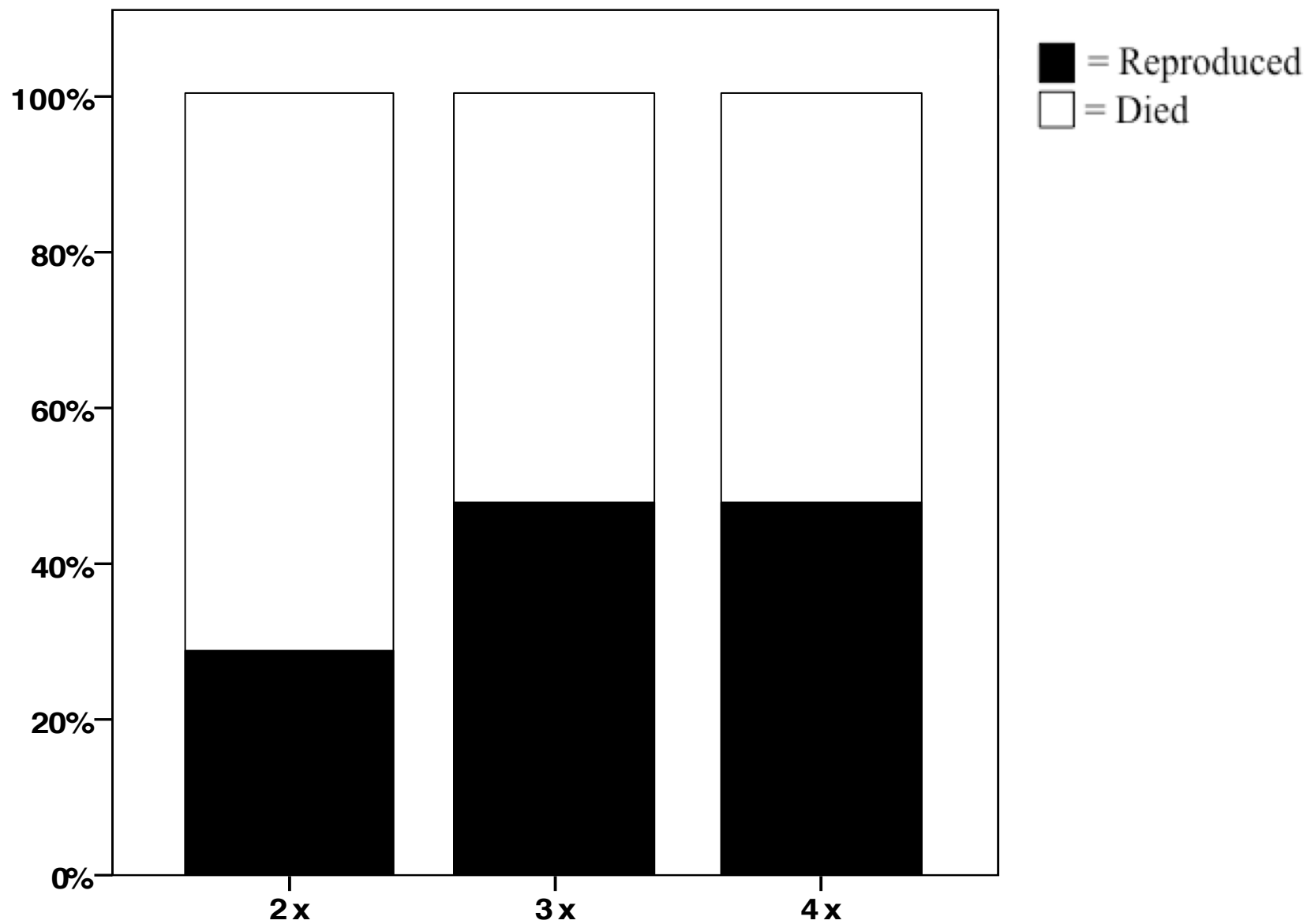

Supplement: Supplementary file 6 — Figure S6. Proportion of 2x, 3x, and 4x snails that reproduced and the proportion of 2x, 3x, and 4x snails that died prior to reproduction. Fisher's exact tests revealed that a significantly higher proportion of sexuals died prior to reproduction than triploid asexuals (P = 0.0034) and relative to tetraploid asexuals (P = 0.0110). There was no significant difference in the proportion of 3x vs. 4x snails that died prior to reproduction (P = 1.0000). [file ECE3-6-765-s006.pdf]
